# Genome-Wide Analysis of *Glycine soja* Response Regulator *GsRR* Genes Under Alkali and Salt Stresses

**DOI:** 10.3389/fpls.2018.01306

**Published:** 2018-09-07

**Authors:** Chao Chen, Ailin Liu, Hao Ren, Yang Yu, Huizi Duanmu, Xiangbo Duan, Xiaoli Sun, Beidong Liu, Yanming Zhu

**Affiliations:** ^1^Key Laboratory of Agricultural Biological Functional Genes, Northeast Agricultural University, Harbin, China; ^2^Crop Stress Molecular Biology Laboratory, Agronomy College, Heilongjiang Bayi Agricultural University, Daqing, China; ^3^Department of Chemistry and Molecular Biology, University of Gothenburg, Gothenburg, Sweden

**Keywords:** *Glycine soja*, alkali stress, salt stress, response regulator, *GsRR2a*

## Abstract

Soil salt-alkalization is a dramatic challenging factor for plant growth. Wild soybean (*Glycine soja*) exhibits a favorable trait of superior tolerance to salt-alkali stress, and recent discoveries show that response regulator family genes are involved in diverse abiotic stresses. Genomic and transcriptomic analyses of all response regulator genes in wild soybean will provide insight into their function in plant stress response. In this study, we identified and characterized a total of 56 *Glycine soja* response regulator (*GsRR*) genes. Phylogenetic analysis suggested that *GsRR* genes could be classified into five subclasses (A1, A2, B1, B2, and C). We further investigated the chromosome locations, gene duplications and conserved domains of the *GsRRs*. Furthermore, the clustering analysis of *GsRR* transcript profiles revealed five different expression patterns under alkali stress. The A1 and A2 subclasses display significantly higher transcriptional levels than the B subclass. In addition, quantitative real-time PCR results verified that the *GsRR* genes were also significantly influenced by salt stress. Notably, *GsRR2a* in the A1 subclass showed opposite expression patterns under salt stress comparing with alkali stress. Moreover, overexpression of *GsRR2a* in *Arabidopsis* significantly improved the tolerance to alkali stress, but not salt stress. These results suggest the important roles of *GsRR* genes in response to salt and alkaline stresses, and also provide valuable clues for further functional characterization of *GsRR* family genes.

## Introduction

Saline-alkali soil is a major factor limiting crop growth, development, and yields. Salt stress in the soil generally causes osmotic stress and ion injury (Zhu, 2003). Alkali stress in the soil is usually characterized by low availability of nutrients, high concentrations of HCO_3_^-^ (bicarbonate) and CO_3_^2-^ (carbonate), and high pH (Yang C.W. et al., 2008; An et al., 2016; Song T. et al., 2017). Owing to hydrolyzation of HCO_3_^-^ and CO_3_^2-^, plants growing on such soils suffer not only sodium toxicity, but also the precipitation Ca^2+^, Mg^2+^, and H_2_PO_4_^-^ (Islam et al., 2011), inhibition of ion uptake (Yang et al., 2007) and disruption of cytoplasmic ion homeostasis (An et al., 2016). Some studies have demonstrated that alkali stress imposes much severer effects than salt stress on plants (Sadras et al., 2003; Shi and Sheng, 2005; Yang et al., 2007), and recent researches also point out a great difference in the physiological adaptive mechanisms of plants responding to alkali stress and salt stress (Borsani et al., 2005; Miller et al., 2010; Rouphael et al., 2017).

With the recent advances in high-throughput sequencing technologies, genes associated with high salinity and alkaline tolerance have been identified on a large scale at a genome-wide level (Jin et al., 2008; Sun et al., 2013; Zhang et al., 2016). The current knowledge of salt-alkali stress transcriptome mainly focuses on salt stress, whereas only limited information concerning alkali stress is available. Wild soybean (*Glycine soja*) exhibits very high adaptability in extreme environments. Our previous studies showed that the wild soybean (G07256) could germinate and set seed even in sodic soil of pH 9.02, and displayed much superior tolerance to 50 mM NaHCO_3_ treatment (Ge et al., 2010), demonstrating that it has developed molecular and physiological mechanisms to adapt itself to this severe condition. Additional, we have identified 3,380 alkaline-responsive genes using RNA sequencing, and also characterized some functional genes under alkaline stress, such as *GsCHX19.3* (Jia et al., 2017), *GsJ11* (Song X. et al., 2017), and *GsTIFY10* (Zhu et al., 2011). Therefore, it is a suitable model organism for studying the molecular mechanisms of plant stress tolerance and a valuable source for characterizing alkali stress responsive genes.

Cytokinins (CKs) are regulators of plant growth and development, and have been shown to control plant responses to salt stress (Tran et al., 2007; Wang et al., 2015). The early response to CKs in *Arabidopsis* involves a multi-step signaling network, in which ARRs (Arabidopsis Response Regulators) play central roles (Jeon and Kim, 2013). The ARRs are divided into three types (type A, B, and C). The type-A ARRs (ARR3-9, ARR15-17, and ARR23) are small proteins with a short receiver domain which contains the phosphorylatable aspartate residue. CK-inducible type-A ARRs act mainly as redundant negative regulators in CK signaling (To et al., 2007). The type-B ARRs (ARR1, ARR2, ARR10-14, and ARR18-21) contain a receiver domain and a large C-terminal region harboring a Myb-like DNA-binding domain for transcriptional activation (Yokoyama et al., 2007). The type-B ARRs are not inducible by CKs, but activate transcription factors that induce transcription of type-A ARRs under CK treatment. Type-C ARRs (ARR22 and ARR24) resemble type-A ARRs, but their expression does not depend on CKs (Horak et al., 2008).

In *Arabidopsis*, the function of *ARRs* has been well suggested to be involved in plant development and signal transduction. *ARR2* is a downstream genes of *ETR1* in ethylene signal transduction (Hass et al., 2004). *ARR3* and *ARR4* play important roles in the circadian control through the CK-independent pathway (Salome et al., 2006). *ARR4* also modulates red light signaling by interacting with phytochrome B (Sweere et al., 2001). Furthermore, studies have demonstrated that *ARRs* play regulatory roles in abiotic stresses. The type-A, -B, and -C *ARRs* are reported to differentially respond to salt stress (Nishiyama et al., 2012). *ARR1* and *ARR12* regulate sodium accumulation in the shoots by controlling the expression of *HKT1* in *Arabidopsis* (Mason et al., 2010). Overexpression of *ARR5*, *ARR7*, and *ARR15* promoted freezing tolerance (Shi et al., 2012). The CK-deficient *Arabidopsis* mutants displayed enhanced drought and salt tolerance, as well as increased ABA sensitivity (Nishiyama et al., 2011). In addition, type-A *ARRs* can act as negative regulators in cold stress signaling through the inhibition of the ABA-dependent pathway (Jeon et al., 2010). However, until now, little is known about the *RR* family genes in response to salt and alkali stresses.

In this study, we identified 56 genes encoding RR proteins in *G. soja* genome. By using phylogenetics to characterize the variations within the *GsRR* family, we found expression of *GsRR* family genes were differentially affected by alkali and salt stresses. We further suggested that one of them, *GsRR2a* played a positive role in response to alkali stress.

## Materials and Methods

### Identification and Characteristics of Response Regulator Family Genes in the *G. soja* Genome

To identify all putative RR family genes in wild soybean, we obtained the *G. soja* genome and proteome sequences, respectively (Jeon et al., 2010; Qi et al., 2014). Because of the limited sequence information for *G. soja*, *G. max* database is used to identify the predicted genes and secondary structure (Zeng et al., 2012). Local BLAST search against *G. soja* proteome was carried out by using the HMM profile (build 2.3.2) of the response regulator domain as query. The HMM profile of receiver domain (ID PF00072) was downloaded from the Pfam database (Punta et al., 2012). The molecular weight and isoelectric point of GsRR proteins were predicted using online software Compute pI/Mw^1^.

### Phylogenetic Tree Construction and Sequence Analysis

To investigate the phylogenetic relationships among GsRR proteins in plants, Clustal X program (Larkin et al., 2007) was used to perform the multiple sequence alignments of all 56 GsRRs from wild soybean and 24 ARRs from *Arabidopsis*. The phylogenetic trees were generated and displayed by using software MEGA 5.0 with the NJ (neighbor-joining) method (Kumar et al., 2008). The MEME^2^ was used to discover conserved motifs of GsRR family proteins. Gene structure maps were generated using GSDS (Gene Structure Display Server)^3^ (Hu et al., 2015). We defined the gene duplication according to the reported standards (Yang S. et al., 2008).

### Plant Materials, Growth Conditions, and Stress Treatments

Seedlings of wild soybean (G07256) were grown in a culture room with the following settings: 60–80% relative humidity, 24–28°C and a light regime of 16 h light/8 h dark. Before sowing, seeds were treated with 98% sulfuric acid for 10–15 min and washed three times with sterile water. Nineteen days after sowing, seedlings were transferred into 1/4 strength Hoagland’s solution with 50 mM NaHCO_3_ or 200 mM NaCl for alkali or salt stress. Equal amounts of leaves and roots were sampled as three biological replicates at 0, 1, 3, 6 h time points after treatments.

### Transcript Level Analysis

In order to analyze the expression profiles of *GsRR* family genes under alkali stress, hierarchical clustering tree based on the transcript data of *GsRR* genes was created with TM4: MeV 4.9 software (Saeed et al., 2003). The transcript data of *GsRRs* in *G. soja* roots subjected to alkali stress was previously obtained in 1 KP project by using transcriptome sequencing, and the data has been deposited in 1KP project^4^.

The expression profiles of *GsRRs* under salt stress were performed by using qRT-PCR (quantitative real-time PCR). The *GAPDH* in *G. soja* was used to normalize all values. Primer sequences of *GsRRs* and *GADPH* are listed in **Supplementary Table S1**. To enable statistical analysis, three fully independent biological replicates were obtained and subjected to qRT-PCR runs in triplicate. Expression levels for all candidate genes were calculated using the 2^-ΔΔCT^ method (Livak and Schmittgen, 2001).

### Transformation of *Arabidopsis*

The CDS region of *GsRR2a* was cloned into the pCAM230035S vector under the control of CaMV35S promoter (primer pairs: 5′-CGGGATCCATGGACACGGACA GCT TCG-3′ and 5′-GCGTCGACTCAATCGGTGCTGGTCA-3′). The pCAM230035S:GsRR2a construct was introduced into *Agrobacterium tumefactions* strain LBA4404 for transformation through floral-dip method (Clough and Bent, 1998). The transformed seeds were selected on 1/2MS medium containing 50 mg L^-1^ kanamycin, and the T_3_ generation overexpression lines were randomly chosen for further studies.

### Phenotypic Analysis Under Alkali and Salt Stresses

The *Arabidopsis* seeds were sterilized as described (Sun et al., 2014). During the early seedling growth stage, the WT and overexpression seeds were sown on 1/2 agar medium supplemented with 0, 7, or 8 mM NaHCO_3_, respectively. The numbers of seedlings with opening and greening leaves were recorded after 12 days. At the adult stage, the 20-day-old WT and overexpression plants grown in nursery pots were irrigated with water or 100 mM NaHCO_3_ every 3 days. Photos were taken after 21 days. The chlorophyll content was detected using the 80% (v/v) acetone extract (Lewinsohn and Gressel, 1983). The malondialdehyde (MDA) content was determined by using a thiobarbituric acid method (Peever and Higgins, 1989). For salt treatment, the WT and overexpression seeds were sown on 1/2 agar medium supplemented with 0 or 150 mM NaCl, respectively. The germination rates were recorded and photos were taken after 6 days.

All experiments were repeated at least three times and the data was subjected to statistical analyses using the SPSS software by Student’s *t*-test.

## Results

### Identification of Response Regulator Genes in *G. soja*

In order to identify GsRR family genes, we used the amino acid sequences of the RR receiver domains (Pfam: PF00072) as queries for BLASTP searches. Sixty-two putative *GsRR* genes were acquired. Then we performed a proteome-wide screen for all putative GsRR by using the Pfam database, four genes were discarded due to the incomplete RR receiver domains and two genes were discarded because of redundancy. Consequently, 56 non-redundant *GsRR* genes were identified, including 19 type-A, 30 type-B, and 7 type-C *GsRRs*. The characteristics of the *GsRR* family genes, including the full CDS length, protein length, molecular weight and pI values are presented in **Table 1**.

**Table 1:** Basic information of the *GsRR* family genes of *G. soja*.

Gene name	Full CDS length (bp)	Protein length (aa)	Molecular weight (Da)	pI	Domain	Similarity with *Arabidopsis*	
*GsRR1a*	735	244	26489.9	5.15	RR	*ARR3*	AT1G59940.1
*GsRR2a*	723	240	26531	4.99	RR	*ARR3*	AT1G59940.1
*GsRR3a*	747	248	28265.8	5.61	RR	*ARR9*	AT3G57040.1
*GsRR4a*	519	172	19577.6	6.44	RR	*ARR9*	AT3G57040.1
*GsRR5a*	615	204	22219.8	8.49	RR	*ARR6*	AT5G62920.1
*GsRR6a*	441	146	16118.8	8.35	RR	*ARR17*	AT3G56380.1
*GsRR7a*	708	235	26476.7	5.32	RR	*ARR9*	AT3G57040.1
*GsRR8a*	636	211	23187.9	8.23	RR	*ARR5*	AT3G48100.1
*GsRR9a*	699	232	26149.4	5.2	RR	*ARR9*	AT3G57040.1
*GsRR10a*	441	146	16003.6	8.34	RR	*ARR17*	AT3G56380.1
*GsRR11a*	615	204	22139.5	7.63	RR	*ARR6*	AT5G62920.1
*GsRR12a*	672	223	24610.3	5.27	RR	*ARR3*	AT1G59940.1
*GsRR13a*	564	187	21226.3	5.62	RR	*ARR9*	AT3G57040.1
*GsRR14a*	636	211	23722	5.38	RR	*ARR9*	AT3G57040.1
*GsRR15a*	540	179	20420.5	6.53	RR	*APRR7*	AT5G02810.1
*GsRR16a*	540	179	20439.5	5.98	RR	*ARR9*	AT3G57040.1
*GsRR17a*	624	207	22844.7	7.59	RR	*ARR8*	AT2G41310.1
*GsRR18a*	669	222	24661.3	5.26	RR	*ARR3*	AT1G59940.1
*GsRR19a*	741	246	27901.5	5.5	RR	*ARR9*	AT3G57040.1
*GsRR1b*	1902	633	69759.6	6.27	RR/Myb-like	*ARR2*	AT4G16110.1
*GsRR2b*	1971	656	72493.4	6.36	RR/Myb-like	*ARR12*	AT2G25180.1
*GsRR3b*	2076	691	76424.3	6.43	RR/CCT motif	*APRR5*	AT5G24470.1
*GsRR4b*	1908	635	71711.8	5.35	RR/Myb-like	*ARR11*	AT1G67710.1
*GsRR5b*	1233	411	45938.8	8.14	RR/Myb-like	*ARR1*	AT3G16857.1
*GsRR6b*	1488	495	55935.2	6.38	RR/Myb-like	*ARR1*	AT3G16857.1
*GsRR7b*	2091	696	76774.2	6.51	RR/Myb-like	*ARR12*	AT2G25180.1
*GsRR8b*	2103	700	77092.2	6.67	RR/CCT motif	*APRR5*	AT5G24470.1
*GsRR9b*	1092	633	69792.6	6.17	RR/Myb-like	*ARR2*	AT4G16110.1
*GsRR10b*	2040	679	74250.5	5.72	RR/Myb-like	*ARR2*	AT4G16110.1
*GsRR11b*	1206	401	45439.9	5.63	RR/Myb-like	*ARR14*	AT2G01760.1
*GsRR12b*	1479	492	54810.5	7.26	RR/Myb-like	*ARR2*	AT4G16110.1
*GsRR13b*	1782	593	66977.2	5.23	RR/Myb-like	*ARR11*	AT1G67710.1
*GsRR14b*	2022	673	73845	6.1	RR/Myb-like	*ARR2*	AT4G16110.1
*GsRR15b*	2097	698	76222	5.57	RR/Myb-like	*ARR12*	AT2G25180.1
*GsRR16b*	2298	765	83325.3	5.85	RR/CCT motif	*APRR7*	AT5G02810.1
*GsRR17b*	1815	604	68074.7	5.42	RR/Myb-like	*ARR11*	AT1G67710.1
*GsRR18b*	1881	626	68442	6.04	RR/CCT motif	*APRR7*	AT5G02810.1
*GsRR19b*	2043	680	75246.4	5.94	RR/Myb-like	*ARR12*	AT2G25180.1
*GsRR20b*	1998	665	73548.4	5.84	RR/Myb-like	*ARR12*	AT2G25180.1
*GsRR21b*	2019	672	73650.7	5.94	RR/Myb-like	*ARR1*	AT3G16857.1
*GsRR22b*	2094	697	76394.6	5.83	RR/Myb-like	*ARR12*	AT2G25180.1
*GsRR23b*	2010	669	73975.8	8.08	RR/Myb-like	*ARR2*	AT4G16110.1
*GsRR24b*	2034	677	73855.1	5.81	RR/Myb-like	*ARR1*	AT3G16857.1
*GsRR25b*	2046	681	75164.1	5.31	RR/Myb-like	*ARR12*	AT2G25180.1
*GsRR26b*	2004	667	73691.4	5.9	RR/Myb-like	*ARR12*	AT2G25180.1
*GsRR27b*	1368	455	51483.7	6.23	RR/Myb-like	*ARR11*	AT1G67710.1
*GsRR28b*	1008	335	38653.4	7.04	RR/Myb-like	*ARR2*	AT4G16110.1
*GsRR29b*	1107	369	41812.9	6.95	RR/Myb-like	*ARR2*	AT4G16110.1
*GsRR30b*	948	315	35909.7	5.39	RR/Myb-like	*ARR2*	AT4G16110.1
*GsRR1c*	399	132	14767	6.51	RR	*ARR24*	AT5G26594.1
*GsRR2c*	342	113	12627.8	9.05	RR	*ARR24*	AT5G26594.1
*GsRR3c*	345	114	12572.2	5.32	RR	*ARR24*	AT5G26594.1
*GsRR4c*	426	141	16341.8	8.7	RR	*ARR24*	AT5G26594.1
*GsRR5c*	351	116	12977.9	5.31	RR	*ARR24*	AT5G26594.1
*GsRR6c*	327	108	11984.9	5.08	RR	*ARR24*	AT5G26594.1
*GsRR7c*	453	150	16958.4	5.56	RR	*ARR24*	AT5G26594.1


### Phylogenetic Analysis of GsRR Proteins

**FIGURE 1:**
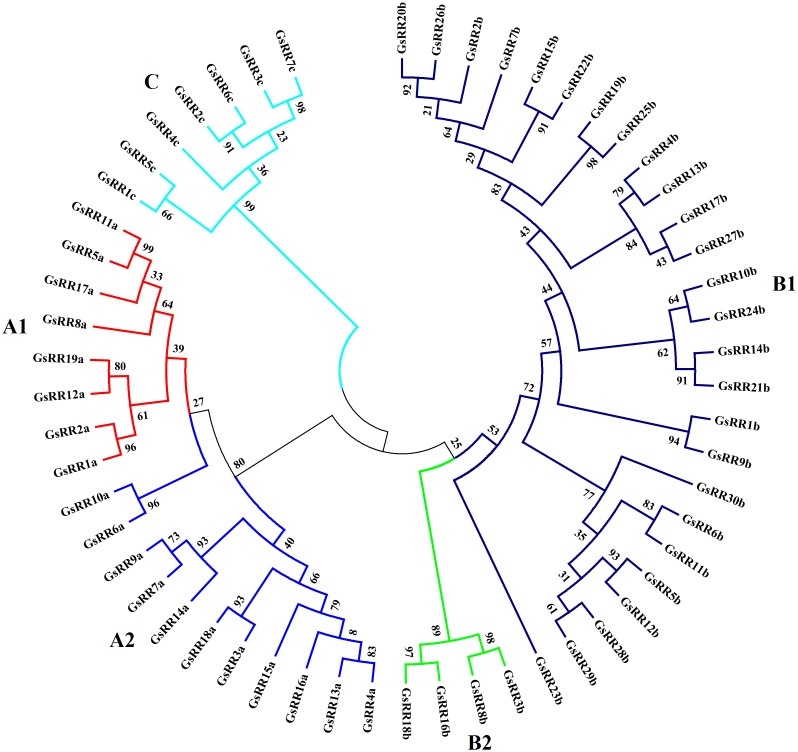
Phylogenetic trees of *GsRR* family genes in *G. soja*. The phylogenetic tree was inferred by MEGA 5.0 with the NJ (neighbor-joining) method after the alignment of protein sequences of the *GsRR* family. The numbers beside the branches represent bootstrap values based on 1,000 replications. *GsRR* family genes were divided into five subclasses and marked by different colors.

To investigate the evolutionary relationship of GsRRs and homologous ARR proteins, we constructed a NJ tree using MEGA 5.0 (**Supplementary Figure S1**). Based on the topology and clade robust bootstrap values, the GsRR proteins were classified into three major classes: type-A, type-B, and type-C. Nineteen GsRRs (GsRR1a to GsRR19a), thirty (GsRR1b to GsRR30b) and seven (GsRR1c to GsRR7c) were clustered into type-A, type-B, and type-C, respectively (**Table 1**). Furthermore, as shown in **Figure 1**, type-A was further divided into two subclasses, designated as A1and as A2. In addition, type-B was also divided into two subclasses (B1 and B2). Most of type-B GsRR proteins belonged to the B1 subclass, only GsRR3b, GsRR8b, GsRR16b, and GsRR18b were clustered into the B2 subclass.

### Physical Locations and Gene Duplications of *GsRRs*

**FIGURE 2:**
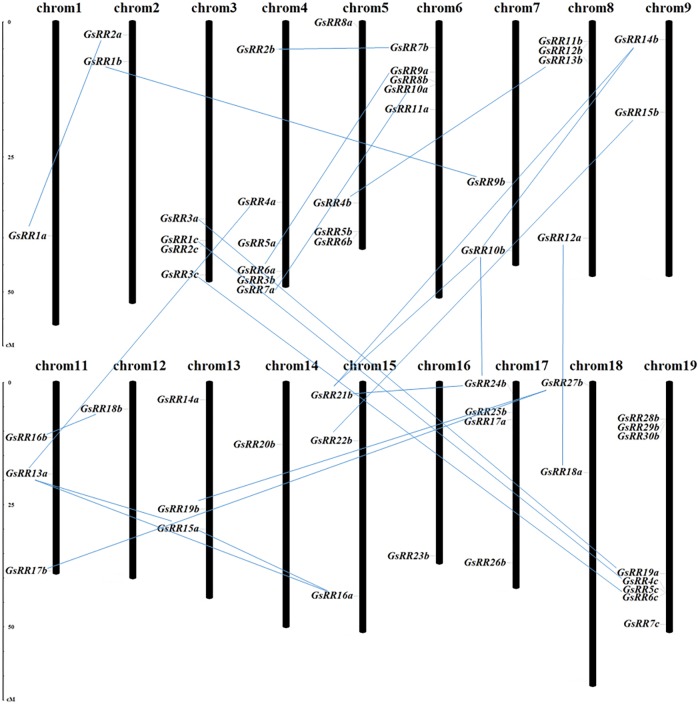
Chromosomal locations and duplications of *GsRR* genes. The scales represent megabases (Mb). The black bars represent the chromosomes. *GsRR* genes distribute on the 18 chromosomes. The paralogous genes are connected by lines.

The potential mechanisms driving the evolution of the *GsRR* family were elucidated by analyzing the gene duplication events. In this study, 56 *GsRR* genes were distributed among 18 chromosomes, with the exception of chromosome 10 and 20 (**Figure 2**). The number of *GsRR* genes in each chromosome differed considerably. For example, 8 *GsRRs* were located on chromosome 19, which chromosomes 1, 12, 14, and 16 only contain one gene, respectively. Using *G. soja* genome duplication information, thirty duplicated gene pairs were identified among 56 *GsRRs*, including three segmental duplication events between chromosomes.

### Conserved Domains and Motifs of *GsRR* Family Genes

**FIGURE 3:**
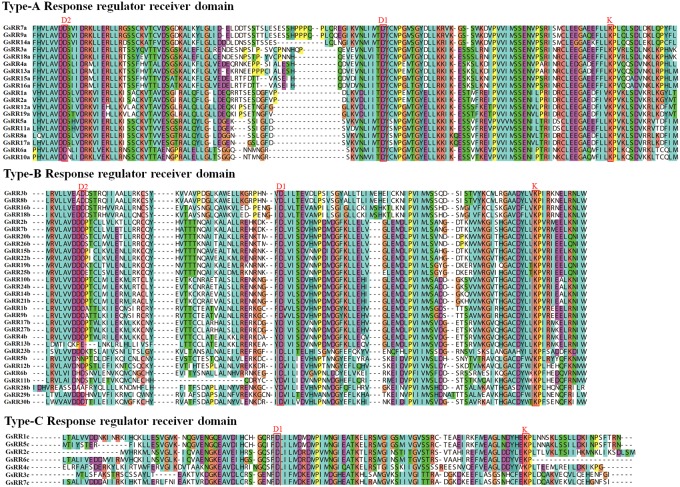
Multiple alignment of *GsRR* family proteins. Multiple sequence alignment shows the RR receiver domains of type-A, -B, and -C. Multiple alignment was performed with Clustal X. The conserved amino acids sites D1, D2, and K are marked.

The modular structure of ARRs has been studied thoroughly in *Arabidopsis* (D’Agostino et al., 2000), which enables us to analyze domain architecture for GsRRs. We identified three conserved domains: a RR receiver domain (PF06200), a Myb-like DNA-binding domain (PF00249) and a CCT motif (PF06203). The RR receiver domain was variable among three types of *GsRR*s (**Figure 3**). The RR receiver domain of type-B GsRRs contained approximately 120 amino acids with three exclusively conserved phosph-accepting amino acids: an invariant D1 site in the center, a D2 site at the N-terminus and a K site at the C-terminus. Compared with type-B, each type-A GsRR has a variable short insertion in the receiver domain and a short C-terminal extension. Type-C GsRRs lost the conserved D2 site in the N-terminus. Remarkably, besides the RR receiver domain, all type-B1 GsRRs contained a C-terminal conserved domain designated as Myb-likes DNA binding domain, which functions importantly in CK responses. In addition, four type-B2 GsRRs contained a CCT motif in the C-terminus. In general, the classification of GsRRs based on their domain composition well supported the phylogenetic results described above.

**FIGURE 4:**
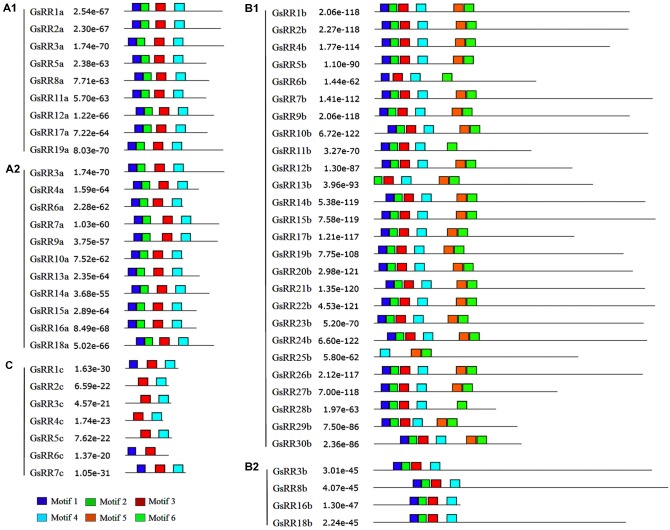
Distribution of conserved motifs in the *GsRR* family members. All motifs were identified by MEME using the full-length amino acid sequences of *GsRR* genes. The *p*-values are showed. Different conserved motifs are indicated by different colors.

To verify the results of domain prediction, the conserved motifs were discovered using MEME on-line tool (Bailey et al., 2009). As shown in **Figure 4** and **Supplementary Figure S2**, when specifying the RR receiver domain, motifs 1, 2, 3, and 4 were found in most type-A and type-B GsRRs. The type-C GsRRs possessed an incomplete RR receiver domain. The Myb-like DNA binding domain, motifs 5 and 6 were distinctively detected in type-B1 members, except GsRR6b, GsRR11b and GsRR28b only included motif 6.

### Expression Patterns of *GsRRs* Genes Under Alkali Stress

**FIGURE 5:**
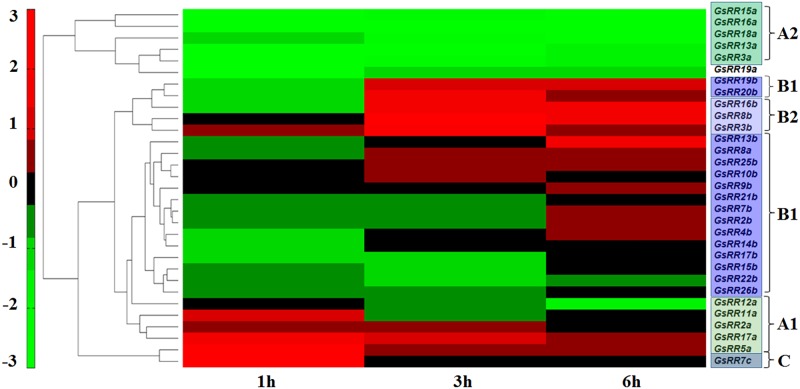
Expression profile of *GsRR* family genes under alkali stress. Expression profiles of *GsRRs* are shown according to the RNA-seq data of wild soybean treated with 50 mM NaHCO_3_. The expression profiles were conducted using Pearson correlation Hierarchical Clustering with TM4: MeV 4.9 software.

The RR family genes are known to be involved in abiotic stress response (Jeon et al., 2010; Mason et al., 2010). The wild soybean G07256 exhibits a much greater tolerance to alkali stress than other plants. Therefore, based on our previous transcriptome data of wild soybean roots under alkali stress (Ge et al., 2010; DuanMu et al., 2015), we performed the expression profiles of *GsRR* family genes using Pearson correlation Hierarchical Clustering with TM4: MeV 4.9 software. The results showed that 31 *GsRR*s were responsive to alkali stress, with distinctive induction dynamics (**Figure 5**). In general, five major expression patterns were unraveled. Five type-A2 *GsRR*s (*15a*, *16a*, *18a*, *13a*, *3a*) and *GsRR19a* formed the first cluster, with significant down-regulation from 1 h to 6 h after alkali stress. Six type-B1 *GsRRs* (*25b*, *10b*, *9b*, *21b*, *7b*, *2b*) and *GsRR8a* showed no obvious change during the treatment. In contrast, type-B GsRRs (*19b*, *20b*, *16b*, *8b* and *3b*) in the third cluster were dramatically up-regulated at 3 h and kept the up-regulated trend in varying degrees until 6 h. The transcript levels of other six *GsRR* genes (*4b*, *14b*, *17b*, *15b*, *22b*, and *26b*) in the fourth cluster were down-regulated and then recovered to the basal levels. It is worth to notice that on the basis of their expression patterns, type-A *GsRRs* were basically separated into two groups, similar with the classification of subclass A1 and A2. The transcript levels of subclass A1 *GsRRs* (*11a*, *2a*, *17a*, and *5a*) were up-regulated at 1 h and then down-regulated at 6 h, which is opposite to subclass A2. These results indicated that *GsRRs* might have different roles in regulating alkali stress response.

### Expression Patterns of *GsRRs* Under Salt Treatment

**FIGURE 6:**
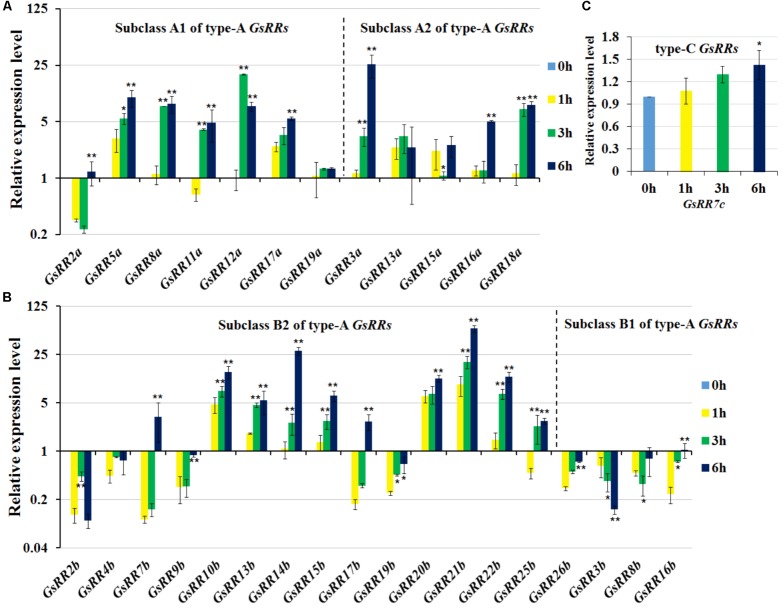
Expression profile of *GsRR* family genes under salt stress. **(A–C)** The expression patterns of *GsRRs* were measured by qRT-PCR analysis with *G. soja* seedlings treated with 200 mM NaCl. Values represent the means of three fully independent biological replicates, and three technology replicates for each. ^∗^*P* < 0.05, ^∗∗^*P* < 0.01 by Student’s *t*-test.

To provide insight into the regulatory mechanisms of *GsRRs* in salt stress, we further analyzed their transcript levels under salt stress using the qRT-PCR analysis. As shown in **Figure 6A**, most type-A *GsRRs* were significantly up-regulated from 1 to 6 h under salt stress. Compared with subclass A2, subclass A1 *GsRRs* responded to salt stress faster and last longer. Unlike alkali stress, among 12 type-A *GsRR*s, only two were down-regulated under salt stress, indicating they responded to salt and alkali stresses in different pathways. For type-B *GsRRs*, three subclass B2 members *GsRR3b*, *GsRR8b*, and *GsRR16b* were down-regulated; eight type-B1 *GsRR*s (*10b*, *13b*, *14b*, *15b*, *20b*, *21b*, and 2*2b*) were up-regulated from 1 to 6 h, seven type-B1 genes were down-regulated at 1, 3, or 6 h (**Figure 6B**). For type-C *GsRRs*, only *GsRR7c* slightly responded to salt stress (less than twofold) (**Figure 6C**).

### QRT-PCR Validation of *GsRR2a* Under Salt and Alkali Stresses

**FIGURE 7:**
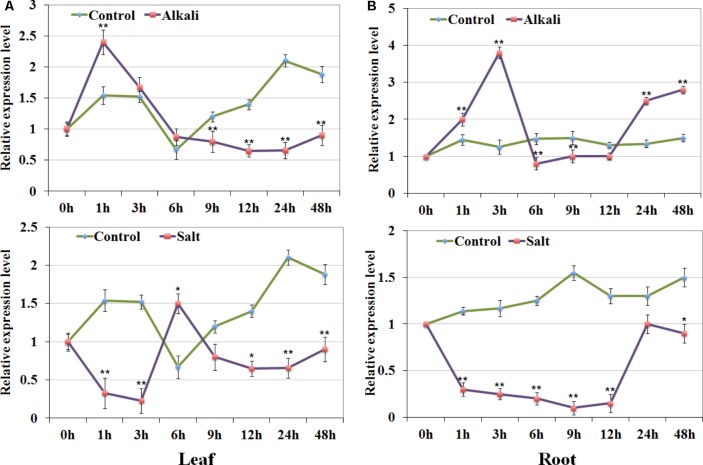
Expression validation of *GsRR2a* in *G. soja.* **(A,B)** Expression levels of *GsRR2a* were detected in root and leaves under salt and alkali stresses using qRT-PCR analysis. Nineteen-day-old of *G. soja* seedlings were submerged into 1/4 Hoagland solution with 50 mM NaHCO_3_ or 200 mM NaCl, respectively. The untreated plants were used as controls. Values represent the means of three fully independent biological replicates, and three technology replicates for each. ^∗^*P* < 0.05, ^∗∗^*P* < 0.01 by Student’s *t*-test.

According to the expression analysis under salt and alkali stress, we focused on one of the type-A1 genes *GsRR2a*, whose expression was strongly induced by alkali stresses, but reduced by salt stress. To confirm this finding, we further detected its expression levels in both roots and leaves of *G. soja s*eedlings under 200 mM NaCl or 50 mM NaHCO_3_ by using qRT-PCR analysis. As shown in **Figure 7**, under alkali treatment, *GsRR2a* showed similar tendencies in leaves and roots. The relative transcript abundance of *GsRR2a* rapidly increased at 1 or 3 h, respectively. Under salt treatment, the transcript abundance of *GsRR2a* was slightly decreased in roots and leaves. These results suggested that *GsRR2a* expression indeed differently responded to alkali and salt stresses.

### Overexpression of *GsRR2a* Improved Tolerance to Alkali Stress in *Arabidopsis*

**FIGURE 8:**
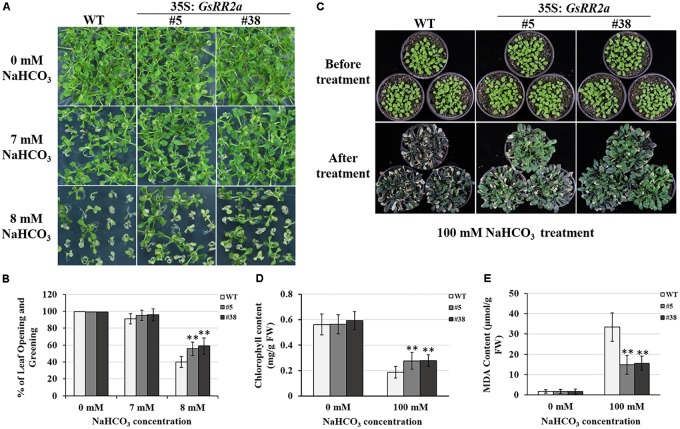
Overexpression of *GsRR2a* in *Arabidopsis* enhances the tolerance to alkali stress. **(A)** The growth performance of WT and overexpression lines on 1/2MS medium containing 0, 7, or 8 mM NaHCO_3_ at early seeding growth stage. **(B)** The numbers of seedlings with opening and greening leaves of WT and overexpression lines. **(C)** The growth performance of WT and overexpression lines before alkali treatment or treated with 100 mM NaHCO_3_ for 16 days. **(D)** The chlorophyll content of WT and overexpression lines. **(E)** The malondialdehyde (MDA) content of WT and overexpression lines. ^∗^*P* < 0.05, ^∗∗^*P* < 0.01 by Student’s *t*-test.

Considering the responsive expression of *GsRR2a* under salt and alkali stresses, we further analyzed the effect of *GsRR2a* overexpression on alkali and salt tolerance. The transgenic lines (#5 and #38) were generated by overexpressing *GsRR2a* in *Arabidopsis*. We firstly performed the early seedling growth assays to determine the tolerance of WT (wide-type) and overexpression lines. Under normal conditions, *GsRR2a* overexpression does not affect plant growth under normal conditions. However, under NaHCO_3_ stress treatment, *GsRR2a* overexpression lines exhibited more seedlings with open and green leaves than WT (**Figures 8A,B**). Furthermore, to evaluate the alkali tolerance at the adult stage, the WT and *GsRR2a* overexpression lines were irrigated with 150 mM NaHCO_3_. After 16 days, the overexpression lines appeared much greener and healthier than WT (**Figure 8C**). In addition, statistical analysis revealed that overexpression lines exhibited higher chlorophyll contents but lower MDA contents than WT (**Figures 8D,E**). In contrast with alkali stress, no significant difference was observed between WT and the overexpression lines in the presence of 150 mM NaCl (**Supplementary Figure S3**). These results suggested that overexpression of *GsRR2a* in *Arabidopsis* could significantly improve the tolerance to alkali stress, but not to salt stress.

## Discussion

Recent studies have reported that the RR family genes regulate plant environmental stress responses through two-component systems (Tran et al., 2010). However, there is limited information about the functions of RR genes in soybean. This study identified all RR family genes in *G. soja* and systematically analyzed their sequences and their responses to salt and alkali stresses. This information may provide useful clues for functional characterization of GsRRs, especially concerning their role in stress tolerance.

In the current study, a total of 56 *GsRRs* were identified in wild soybean genome. These *GsRRs* were classified into five subclasses according to their phylogeny, which is consistent with previous reports in *Arabidopsis* and rice (D’Agostino et al., 2000; Jain et al., 2006). Interestingly, there were more *GsRRs* containing Myb-like DNA domain in type-B than type-A, which may attribute to gene duplication events. The *Arabidopsis* genome contains almost the same number of type-A and type-B *ARRs*. By contrast, the maize genome contains more type-A *ZmRRs* (Chu et al., 2011). These indicated that type-B RRs containing the Myb-like DNA binding domain might play more important roles in dicots. Different from *Arabidopsis*, type-A *GsRRs* are further divided into two subclasses (8 members in subclass A1 and 11 in subclass A2), which suggests possible divergence of their functions during evolution. Moreover, four type-B *GsRR*s (3b, 8b, 16b, and 18b) were designed as subclass B2. Subclass B2 members are also called the pseudo-response regulators, which are the circadian clock component proteins in *Arabidopsis*. They contain a receiver-like domain lacking the conserved phosphoacceptor aspartic acid residue, and a CCT motif responsible for transcriptional repression (Chu et al., 2011; Wang et al., 2013).

The motif distribution analyzed by MEME was basically consistent with the phylogenetic analysis. *GsRRs* in each individual subclass usually shared subclass-specific motifs. Besides, different types of *GsRRs* contained different numbers of exons (**Supplementary Figure S4**). For example, type-A *GsRRs* contained five exons, whereas type-B GsRRs contained four to nine exons. The different numbers of exons possibly shared evolutionary and structural differences.

Roots are the first point perceiving the underground environment stress. To explore the possible functions of RRs under alkali stress, we investigated the transcript levels of *GsRR*s in wild soybean roots. From their expression profiles, we observed five type-A2 *GsRR*s (*15a*, *16a*, *18a*, *13a*, *3a*) showed the same expression pattern under alkali stress, where they were significantly and continuously down-regulated upon the NaHCO_3_ treatment. This result suggested these co-expressed type-A2 *GsRR*s might function negatively in alkali stress responses. Interestingly, other five type-A1 genes *GsRR12a*, *GsRR11a*, *GsRR2a*, *GsRR17a*, and *GsRR5a* were also closely clustered and showed co-expression in roots. This further implies the functional redundancy among GsRRs, and functional divergence between type-A1 and type-A2 in plant tolerance to alkali stress. Moreover, *GsRR14b*, *GsRR15b*, *GsRR17b GsRR21b*, *GsRR22b*, and *GsRR26b* in subclass B1 were strongly down-regulated at 1 h or 3 h, while other subclass B1 *GsRRs* were significantly up-regulated at 3 h or 6 h. The difference among subclass B1 members in alkali stress responses may be resulted from different upstream or downstream regulatory elements or factors, which indicated diversified functions within the same subclass.

The great difference in expression patterns of *GsRRs* to alkali and salt stresses bring us to consider there might be other regulatory mechanism and signal pathway in alkali stress. As we know, that salt stress involves osmotic stress and ion injury, and salinity tolerance in plants largely contributed by Na^+^ exclusion (Yamaguchi et al., 2013). Actually, it has been pointed out that high HCO_3_^-^ can diminish leaf area and length, decrease shoot biomass, and reduce the photosynthetic rate. However, the molecular mechanism of plant response to alkali stress is rarely known. Considering the important roles of RR proteins in CK signaling, the induction of *GsRRs* expression by salt and alkali stress provides a molecular link between stress and CK signaling. Moreover, *GsRR2a*, the homologous gene of *ARR3*, could enhance plant tolerance to alkali stress, but not to salt stress. One possible reason is that *GsRR2a* was up-regulated under alkali stress which indicated that this gene may be as a positive regulator of plant tolerance to alkali stress. However, *GsRR2a* exhibited the opposite expression pattern to salt and alkali stresses, which implied that *GsRR2a* may participate in different signaling pathways under alkali and salt stresses. In total, these results support the different mechanisms for alkali and salt stresses, and also provide a foundation for future work to elucidate the function of GsRR family genes.

## Conclusion

In summary, we identified 56 *GsRR* genes, which could be classified into three types (five subclasses). *GsRR* were distributed among 18 chromosomes with gene duplications. Moreover, *GsRR* genes exhibited different expression patterns under alkali and salt stresses. Furthermore, overexpression of *GsRR2a* in *Arabidopsis* significantly improved the tolerance to alkali stress. In total, our results showed that *GsRRs* play crucial roles in plants responses to alkali and salt stresses. These results provided a foundation for further functional characterization of *GsRR* family genes.

## Author Contributions

CC, AL, HR, YY, HD, and XD performed the experiments and analyzed data. CC and AL wrote the manuscript. BL interpreted data and revised the manuscript. DZ, XS, and YZ provided ideas and designed the research. All authors have read and approved the final manuscript.

## Conflict of Interest Statement

The authors declare that the research was conducted in the absence of any commercial or financial relationships that could be construed as a potential conflict of interest.

## Footnotes

1 http://au.expasy.org/tools/pi_tool.html

2 http://meme-suite.org/

3 http://gsds.cbi.pku.edu.cn/

4 http://www.onekp.com/samples/list.php

## Supplementary Material

FIGURE S1 Phylogenetic trees of *GsRR* family of *G. soja* and *Arabidopsis*. Neighbor-joining phylogenetic tree of the response regulator members in *G. soja* and *Arabidopsis*. The tree was inferred by MEGA 5.0 with the neighbor-joining method after the alignment of the full-length amino acid sequences of the 56 *G. soja* genes and 24 *Arabidopsis* genes. The numbers beside the branches represent bootstrap values based on 1,000 replications. The scale bar corresponds to 0.1 estimated amino acid substitutions per site.

FIGURE S2 Distribution of conserved motifs. All motifs were identified by MEME using the complete amino acid sequences of *GsRR* genes.

FIGURE S3 Overexpression of *GsRR2a* in *Arabidopsis* did not affect the tolerance to salt stress. The WT and overexpression lines are grown on medium containing 0 or 150 mM NaCl. The germination rates were recorded and photos were taken after 6 days.

FIGURE S4 Structure analysis of *GsRR* genes using GSDS online tools. The UTRs (upstream/downstream sequences), exons and introns are shown with light blue boxes, yellow boxes, and black lines, respectively.

TABLE S1 Gene-specific primers of *GsRR* family used for q-RT PCR assays.
