# Supplementary figures and images for: Genome-Wide Analysis of Glycine soja Response Regulator GsRR Genes Under Alkali and Salt Stresses
Source: Front Plant Sci. 2018 Sep 7;9:1306. doi: 10.3389/fpls.2018.01306 (PMC6137175; doi:10.3389/fpls.2018.01306)

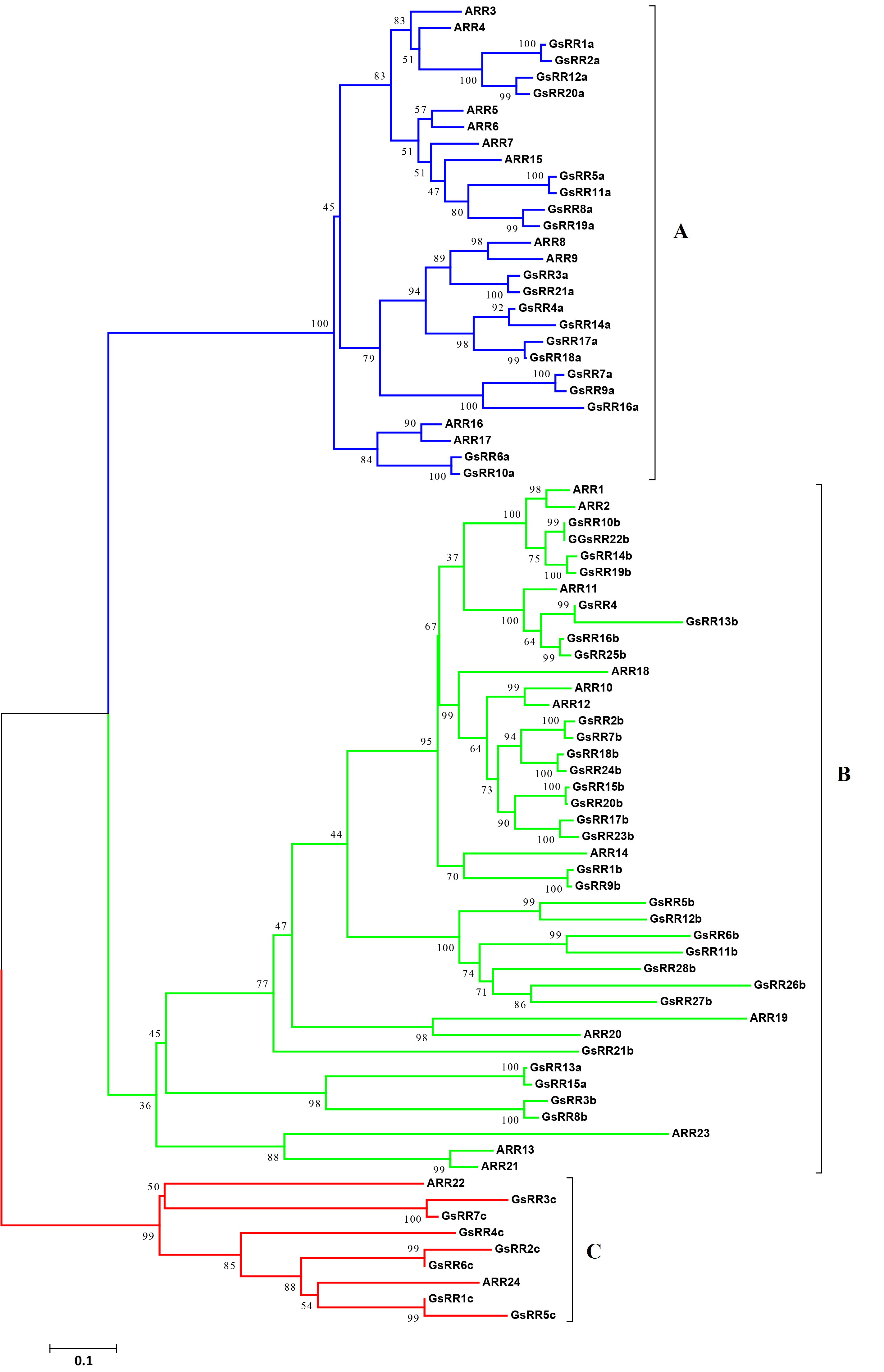

Supplement: FIGURE S1 — Phylogenetic trees of GsRR family of G. soja and Arabidopsis. Neighbor-joining phylogenetic tree of the response regulator members in G. soja and Arabidopsis. The tree was inferred by MEGA 5.0 with the neighbor-joining method after the alignment of the full-length amino acid sequences of the 56 G. soja genes and 24 Arabidopsis genes. The numbers beside the branches represent bootstrap values based on 1,000 replications. The scale bar corresponds to 0.1 estimated amino acid substitutions per site. [file Image_1.TIF]
