# Supplementary material for: Genome-Wide Analysis of Glycine soja Response Regulator GsRR Genes Under Alkali and Salt Stresses
Source: Front Plant Sci. 2018 Sep 7;9:1306. doi: 10.3389/fpls.2018.01306 (PMC6137175; doi:10.3389/fpls.2018.01306)
Supplement: TABLE S1 — Gene-specific primers of GsRR family used for q-RT PCR assays. [file Table_1.DOCX]

**Table S1 Gene-specific primers of *GsRR* family used for q-RT PCR assays**

| Gene name | Primer sequence (5'-3') |
| --- | --- |
| *GAPDH* | Forward: GACTGGTATGGCATTCCGTGT |
|  | Reverse: GCCCTCTGATTCCTCCTTGA |
| *GsRR2a* | Forward: GGTCCTTCTCTGCTCGTCCA |
|  | Reverse: GCCGTTGATGACAGCCTCG |
| *GsRR3a* | Forward: GGCTCCTTCCTCCAAACATC |
|  | Reverse: GAGTTTCTGGGGTTGCGTG |
| *GsRR2b* | Forward: TCCAAGCATAAAAGGGGGC |
|  | Reverse: GTCTTTCTCACTCCTCGCTGCA |
| *GsRR5a* | Forward: TTTTCTCCATCCAACCCCAG |
|  | Reverse: GGCGATGTTTTCAGGCAGG |
| *GsRR3b* | Forward: TTCAACCAACAGCACCCTCA |
|  | Reverse: TGGAAGTGGGAGCAAGGC |
| *GsRR8a* | Forward: CAGGCACCTATCAATTCGGG |
|  | Reverse: TTCAGTATCTGGGGTTGGACG |
| *GsRR4b* | Forward: AGATCCTATTGAACTCACAGG |
|  | Reverse: GGATAGCCTCAGCCAGTTGC |
| *GsRR7b* | Forward: CAGCGCCAACGCCTTTA |
|  | Reverse: GAATGATTCCGGCGACGG |
| *GsRR8b* | Forward: GAGAGAATGCCCTGGCTGG |
|  | Reverse: ACATCTCACGCATCGCTACTG |
| *GsRR11a* | Forward: CGATCACCTTGCGGTCCAC |
|  | Reverse: CACCCTTTTCCCCTAACTTCA |
| *GsRR9b* | Forward: ACGGTAACGGCAGCGAATA |
|  | Reverse: CGATTCCTCGGCTATGCTC |
| *GsRR10b* | Forward: CTCCACGGACCAAACAACC |
|  | Reverse: GCGAATGAAGGCAGTTGGA |
| *GsRR13b* | Forward: AAAAGAATACCCCAGCCCC |
|  | Reverse: GATCGGTCCTGCTCTTCCC |
| *GsRR12a* | Forward: GCTTGCAGCTGTAGTGCCTAC |
|  | Reverse: CACCACTCCCATCGTCACC |
| *GsRR14b* | Forward: AATTAGGAAGAGAACGGGCAG |
|  | Reverse: CTGAAACAGCAAGAAGGCGT |
| *GsRR15b* | Forward: CATGCCCCATAGAACTTGTCAC |
|  | Reverse: GAGCTGAGATGCCAAGAGGG |
| *GsRR16b* | Forward: GCTCCTTCCTAGGTGAACG |
|  | Reverse: CGACATTCCTCAATAGCACCA |
| *GsRR13a* | Forward: CCTTGCTGGGACATTCTCTGA |
|  | Reverse: GAGCCTCCTCCCTGTATTGC |
| *GsRR17b* | Forward: TTTCTTCTGACTCCAGAGGGG |
|  | Reverse: TCACAGTCCACCATAGCGAAA |
| *GsRR19b* | Forward: CACCTCTCATCACCCGCTC |
|  | Reverse: TGCTTCGCAAATGCCAGT |
| *GsRR15a* | Forward: GGGAGTCTGCTTGAGGGTAAA |
|  | Reverse: CATGCAAAAGGGATAAAGGG |
| *GsRR20b* | Forward: TCGGTTCGCCACACAGC |
|  | Reverse: GGAGGAGAACGAGGATGGC |
| *GsRR21b* | Forward: ACAGGAGCATTGACTCGCATA |
|  | Reverse: GCTGGTGTGCCTATGCCTC |
| *GsRR22b* | Forward: GATACTGGCATTTGCGAAGG |
|  | Reverse: AAGATGAGGGCTGTGACCGT |
| *GsRR16a* | Forward: CACTACCAAGCAAGCGAGAGA |
|  | Reverse: TGGAAGAAATCCATTCCCCA |
| *GsRR24b* | Forward: ATCAGATCCTCCTGCCCATAA |
|  | Reverse: TACACAACAGACCGGCCAAA |
| *GsRR17a* | Forward: GCCTTCTTCGTGGATAATGGT |
|  | Reverse: TGGAGGAAGGAGCAGAGGAT |
| *GsRR26b* | Forward: TGGCTTCATCCAAGACAACC |
|  | Reverse: TCGTTTTCCCTATGTCTTCCC |
| *GsRR18a* | Forward: GAGGGTATTTTCTGCTCGTCC |
|  | Reverse: CCTCAAACAGAACCAATCCGA |
| *GsRR19a* | Forward: TGGGATTGAGAGAGGAAGAGC |
|  | Reverse: CCTCTCTCAATCCCATCTCCC |
| *GsRR7c* | Forward: CGTAGATGTTTTGTCGCCTCG |
|  | Reverse: TAGAGGCAAAAACGGTTAGGG |
